# New Computation of Resolving Connected Dominating Sets in Weighted Networks

**DOI:** 10.3390/e21121174

**Published:** 2019-11-29

**Authors:** Adriana Dapena, Daniel Iglesia, Francisco J. Vazquez-Araujo, Paula M. Castro

**Affiliations:** Department of Computer Engineering, Campus de Elviña, Universidade da Coruña, 15071 A Coruña, Spain; daniel.iglesia@udc.es (D.I.); fjvazquez@udc.es (F.J.V.-A.); paula.castro@udc.es (P.M.C.)

**Keywords:** complex networks, connected dominating set, graph entropy, resolving set, vertex importance

## Abstract

In this paper we focus on the issue related to finding the resolving connected dominating sets (RCDSs) of a graph, denoted by *G*. The connected dominating set (CDS) is a connected subset of vertices of *G* selected to guarantee that all vertices in the graph are connected to vertices in the CDS. The connected dominating set with minimum cardinality, or minimum CDS (MCDS), is an adequate virtual backbone for information interchange in a network. When distinct vertices of *G* have also distinct representations with respect to a subset of vertices in the MCDS, it is said that the MCDS includes a resolving set (RS) of *G*. With this work, we explore different strategies to find the RCDS with minimum cardinality in complex networks where the vertices have different importances.

## 1. Introduction

A weighted network can be modeled as a graph where the vertices have weights representing the metric of some relevant parameter. The identification of such relevant vertices is important for better control of disease spreading [1], design of marketing strategies [2], optimization of limited resource allocation [3], and so on.

In weighted networks, the concept of entropy allows one to establish a measure of the information in the relational structure. The first definitions of graph entropy were presented by Rasherky [4], Trucco [5], and Mowshowitz [6,7,8,9]. From those works to the present, a large number of definitions have been proposed in the literature. We recommend consulting the survey presented in [10].

In the context of wireless ad hoc sensor networks, in which the vertices have different weights to reflect, for example, residual energy or capabilities for a certain task, some specific algorithms based on graph theory have been proposed for the computation of sets of vertices needed to build a virtual backbone or fault tolerance. For such applications, recent papers have implemented the known as connected dominating sets (CDSs) and the resolving sets (RSs) [11].

A CDS is a subset of vertices constituting a connected induced subgraph such that every vertex in the network is either in the CDS or has a neighbor in it. The CDS with minimum cardinality or minimum CDS (MCDS) is a natural candidate to be used for the information interchange in any type of network. Thus, its utilization for defining virtual backbone infrastructure in ad hoc wireless networks has been addressed in the last decade [12,13,14,15]. In Ref [16], we have proposed an algorithm based on constructing the CDS for any type of graph taking into account both weights and degrees of vertices. We have also shown the relationship between the MCDS computation for weighted graphs and some theoretical concepts about metrics, like error probability, entropy, or entropy variation.

On the other hand, the RS is a subset of vertices such that each vertex in the network has distinct representation considering as a metric the distance to other vertices in the RS. Both RS and minimum RS (MRS) have been presented in [17] to describe the use of these ideas when working with sonar and coast guard stations. Similar ideas have also been applied for identification in wireless networks [18] or feature extraction in classification algorithms [19].

Recently, research has combined CDS with RS by means of defining the resolving domination in graphs [20,21]. The cardinality of a minimum resolving dominating set has been introduced in [20] to indicate the minimum number of vertices needed to have a dominating set containing an RS. This idea has been exploited in [21] additionally considering connectivity between vertices in the dominating set. This work also presents the minimum cardinality of the RCDS for standard graphs. However, it is not focused on the way of obtaining that RCDS.

The main contribution of this paper compared to [20,21] is the search of this RCDS throughout different algorithms. In this sense, we consider three different approaches according to their respective types of RSs: the first one, referred to as contained-MRS (c-MRS), considers an MCDS that contains some MRS; the second scenario, referred to as non-contained-MRS (nc-MRS), considers an MCDS that does not contain any MRS, and the last one, referred to as contained-RS (c-RS), in which an MCDS does not contain any c-MRS, although an RS can be possible. Based on those graph types, we propose several methods and show examples of performance for standard graphs, like paths, cycles, or grids.

The identification of the RCDS has many applications. For instance, in this paper, we propose the utilization of RCDS to determine an adequate set of vertices that allows us to build a virtual backbone and to uniquely identify all network vertices. The construction of a CDS or an RS for wireless networks has been separately addressed in the literature, but as far as we know, the combined issue has not been previously studied.

This paper is organized as follows. Section 2 reviews some definitions for the understanding of this work. Section 3 introduces the concepts of c-MRS, nc-MRS and c-RS showing their computation using simple well-known graphs. This section also presents three algorithms for the computation of an RCDS considering the above mentioned definitions. Some results are shown in Section 4 and the concluding remarks are briefly made in Section 5.

## 2. Previous Definitions

A graph G=(V;E) consists of a set of vertices, denoted by *V*, and of a set of edges, denoted by *E*. The vertices correspond to the objects to be modeled, while the edges indicate some relationship between pairs of these objects. We assume that *G* has no loops, nor multiple or directed edges, and there is a path between any pair of its vertices. The distance d(u,v) between two vertices *u* and *v* of a graph *G* is the minimum length of the paths connecting them (i.e., the number of hops between *u* and *v*).

### 2.1. Dominating and Resolving Sets

**Definition** **1.** *Connected Dominating Set (CDS). Given a graph G, a dominating set D is a subset of the vertices of G, such that each vertex in G is either in D or is adjacent to a vertex in D. This set is a CDS if each vertex in D is connected with at least one vertex in D.*

The concept of connected domination number, i.e., $\gamma_{c}\left(G\right)$, was introduced by Sampathkumar and Walikar [22] to denote the minimum cardinality of a dominating set in *G*. The CDS with minimum cardinality is referred to as MCDS.

**Definition** **2.** *Resolving Set (RS). A set W of k vertices is a resolving set of G if r(u|W)=r(v|W) implies that u=v, where*

(1) $$\begin{array}{c} r\left(u|W\right)=(d\left(u,w_{1}\right),d\left(u,w_{2}\right),\ldots ,d\left(u,w_{k}\right)). \end{array}$$

The term $\gamma_{r}\left(G\right)$, introduced by Slater [17], denotes the minimum cardinality of the resolving sets of a graph *G*. The RS with minimum cardinality is referred to as MRS.

Robert et al. in [20] introduced the concept of resolving domination in graphs and Naji et al. in [21] extended these ideas to CDS, as defined in the following:

**Definition** **3.** *Resolving Connected Dominating Set (RCDS) [21]. A set D of vertices of G that is both resolving and connected dominating is a resolving connected dominating set.*

The minimum cardinality of an RCDS is referred to as the resolving connecting domination number and denoted by $\gamma_{rc}\left(G\right)$. The set of vertices constitutes the minimum RCDS (MRCDS). In the following section, we will show some examples where the MRS is not included in the MRCDS.

**Proposition** **1.** *This proposition indicates some results derived from $\gamma_{rc}\left(G\right)$ for several well-known types of graphs [21].*

*1.*  *Complete graph: $\gamma_{rc}\left(K_{n}\right)=n-1$, for n≥2.*
*2.*  *Path graph: $\gamma_{rc}\left(P_{n}\right)=n-2$, for n≥4.*
*3.*  *Cycle graph: $\gamma_{rc}\left(C_{n}\right)=n-2$, for n≥3.*
*4.*  *If G is a connected graph of order n≥2 and diameter d, then*
  (2) $$\begin{array}{ccc} \gamma_{rc}\left(G\right)\geq f\left(n,d\right) & = & min\left\{k+\sum_{i=1}^{k}\left(\begin{array}{c} k\\ i \end{array}\right){\left(d-1\right)}^{k-i}\right\}. \end{array}$$

### 2.2. Entropy and Entropy Variation

The classical definition of entropy graphs arises from Shannon’s entropy formula [23] considering a weighted network where each vertex has a certain vertex reliability. The vertex reliability can be computed using centrality measures (degree, betweenness, etc.) or specific capabilities of a vertex for a specific task and it can be computed using global or local information [24,25].

**Definition** **4.** *Entropy of a graph. Given certain vertex reliability, denoted by f, we obtain*

(3) $$p\left(v\right)=\frac{f(v)}{\sum_{v\in V}f\left(v\right)}.$$

*As $\sum_{v\in V}p\left(v\right)=1$, p(v) can be interpreted as a probability mass function so that the entropy of a graph G can be defined as follows:*

(4) $$\begin{array}{cc} I_{f}\left(G\right) & =-\sum_{v\in V}p\left(v\right)\log_{2}\left(p\left(v\right)\right)=-\sum_{v\in V}\frac{f(v)}{\sum_{v\in V}f\left(v\right)}\log_{2}\left(\frac{f(v)}{\sum_{v\in V}f\left(v\right)}\right)\\ \; & =\log_{2}\left(\sum_{v\in V}f\left(v\right)\right)-\sum_{v\in V}\frac{f(v)}{\sum_{v\in V}f\left(v\right)}\log_{2}f\left(v\right). \end{array}$$

In Ref [26], the entropy defined by Equation (4) is interpreted as a measure of the amount of information encoded in the network structure and the entropy variation is introduced to give an idea of such an influence.

**Definition** **5.** *Entropy Variation [26]. The importance of a vertex $v_{i}$ in a graph G with information function f is defined as the Entropy Variation under the removal of vertex $v_{i}$*

(5) $$\begin{array}{ccc} EV(v_{i}) & = & I_{f}\left(G\right)-I_{f}\left(G_{v_{i}}\right). \end{array}$$

*Here, $G_{v_{i}}$ is the modified graph under the removal of $v_{i}$, i.e.,*

(6) $$\begin{array}{c} G_{v_{i}}=\left(V-v_{i},E-\left\{u,w\;|u=v_{i}\;\;or\;\;w=v_{i}\right\}\right). \end{array}$$

## 3. Principles and New Methods

The purpose of this paper is to propose and compare different methods to compute the RCDS. Firstly, we will establish a classification of MRS taking into account if the MRS is contained in the MCDS or not. For this classification, we will show some examples considering simple graphs. Finally, we will present the algorithms for RCDS computation.

### 3.1. Definitions

**Definition** **6.** *Contained-MRS (c-MRS). Let $W_{m}$ be an MRS with cardinality $\gamma_{r}\left(G\right)$ and $D_{m}$ be an MCDS with cardinality $\gamma_{c}\left(G\right)$. Then, if $W_{m}\subseteq D_{m}$, $W_{m}$ is a c-MRS.*

**Definition** **7.** *Non-contained-MRS (nc-MRS). Let $W_{m}$ be an MRS with cardinality $\gamma_{r}\left(G\right)$ and $D_{m}$ be an MCDS with cardinality $\gamma_{c}\left(G\right)$. Then, if $W_{m}⊈D_{m}$, $W_{m}$ is a nc-MRS.*

**Definition** **8.** *Contained-RS (c-RS). Let W be an RS with cardinality $|W|>\gamma_{r}\left(G\right)$ and $D_{m}$ be an MCDS with cardinality $\gamma_{c}\left(G\right)$. Then, if $W\subseteq D_{m}$, W is a c-RS but not a c-MRS.*

Since the calculation of an RCDS is an NP-complex problem, we propose to find the RCDS as the union of the RS and the MCDS. Then, we have the following classes

1. For a c-MRS, it satisfies $W_{m}⋃D_{m}=D_{m}$, and $\gamma_{rc}\left(G\right)=\gamma_{c}\left(G\right)$. As a consequence, we have an MRCDS.
2. For a c-RS, it satisfies $W⋃D_{m}=D_{m}$, and $\gamma_{rc}\left(G\right)=\gamma_{c}\left(G\right)$. As a consequence, we have an MRCDS.
3. For an nc-MRS and $W_{m}\cap D_{m}=\emptyset$, it satisfies $|W_{m}⋃D_{m}|=\gamma_{c}\left(G\right)+\gamma_{r}\left(G\right)$. In such a case, we can not determine if this is an MRCDS or not.
4. For an nc-MRS and $W\cap D_{m}\neq \emptyset$, it satisfies $|W⋃D_{m}|<\gamma_{c}\left(G\right)+\gamma_{r}\left(G\right)$. In such a case, we can not determine if this is an MRCDS or not.

### 3.2. Examples

Now we will show some examples to illustrate those previous definitions.

**Figure 1:**
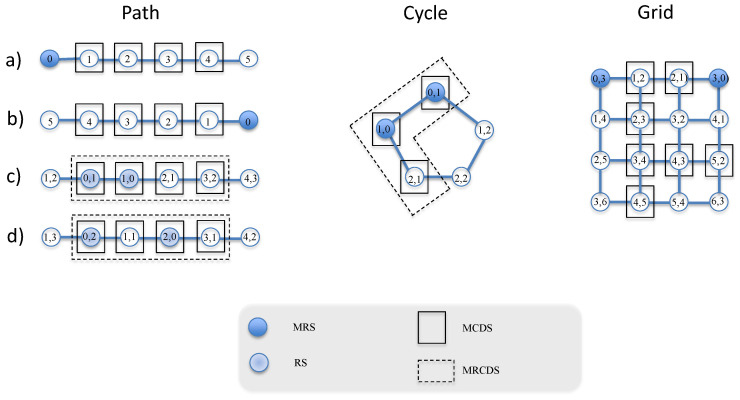
Examples of graphs: a path graph with n=6 vertices (**left**), a cycle graph with n=5 vertices (**center**), and a grid path with n=16 vertices (**right**).

- **Path graph.**Figure 1 (left) shows a path graph with n=6 vertices i.e., $P_{6}$. We can see that there exists only one MCDS, which is constituted by all vertices except corners. The cardinality is $\gamma_{c}\left(P_{6}\right)=n-2=4$. In contrast, the MRS has one corner i.e., $\gamma_{r}\left(P_{6}\right)=1$. Note that the MCDS does not contain any MRS i.e., the path graph has two nc-MRS. The top graphs (a) and (b) of this figure show the two RCDSs obtained with $W_{m}⋃D_{m}$ and their corresponding labels obtained computing Equation (1) for each vertex of the path graph. Note that the cardinality of this RCDS is $|W_{m}⋃D_{m}|=\gamma_{c}\left(G\right)+\gamma_{r}\left(G\right)=n-1=5$.
  In a path graph, it is possible to identify the RS formed by two vertices contained in the MCDS. For example, the path graph with $P_{6}$ has six c-RS. The bottom subfigures (c) and (d) of Figure 1 shows two c-RSs and their corresponding MRCDSs, which is equal to the MCDS. In this case, we have that $\gamma_{rc}\left(P_{6}\right)=\gamma_{c}\left(P_{6}\right)=4$.
  Comparing the cardinality of the RCDS obtained from (a) and (b) to that from (c) and (d), we conclude that $W_{m}⋃D_{m}$ does not provide an MRCDS.
- **Cycle graph.**Figure 1 (center) shows a cycle graph with n=5 vertices i.e., $C_{5}$. The MCDS has a cardinality of $\gamma_{c}\left(C_{5}\right)=n-2=3$, and the MRS has $\gamma_{r}\left(C_{5}\right)=2$. Note that it is possible to find three different c-MRS. The corresponding MRCDS is the MCDS with cardinality $\gamma_{rc}\left(C_{5}\right)=\gamma_{c}\left(C_{5}\right)=3$.
- **Grid graph.**Figure 1 (right) shows a grid path with n=16 vertices i.e., $G_{16}$. The MCDS has a cardinality of $\gamma_{c}\left(G_{16}\right)=7$ [27] and the MRS is formed by opposite corners [28]. As a consequence, $W_{m}\cap D_{m}=\emptyset$ and we can obtain an RCDS by $W_{m}⋃D_{m}$ with cardinality $\gamma_{rc}\left(G\right)=\gamma_{c}\left(G\right)+\gamma_{r}\left(G\right)=9$, although we are not able to determine if it is an MRCDS or not.

### 3.3. Computation of an MRS

**Figure 2:**
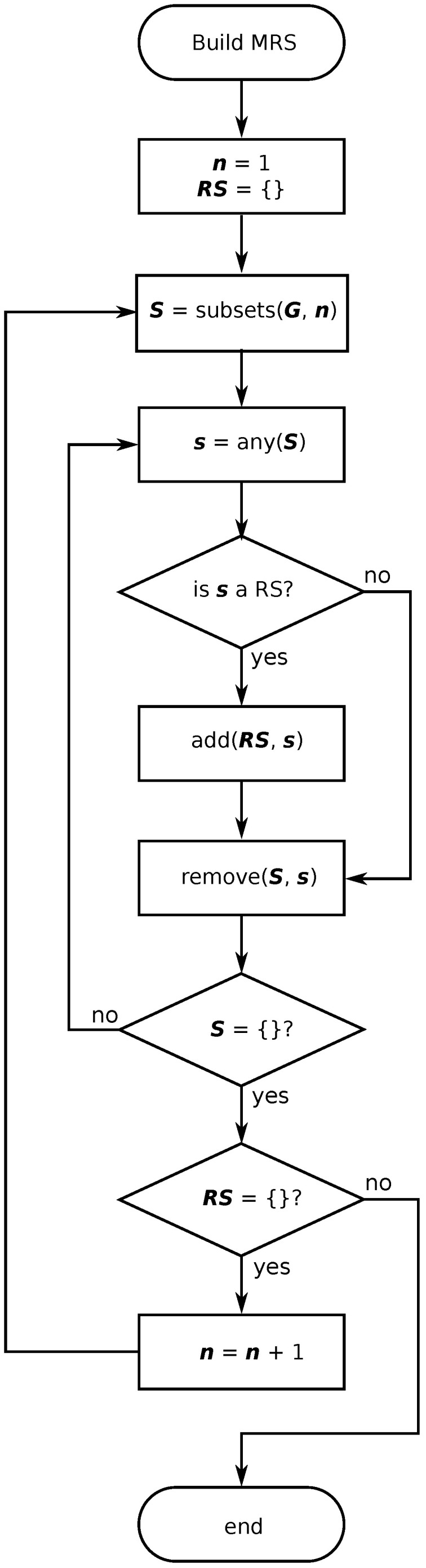
Flowchart for the computation of the minimum resolving set (MRS).

Considering a graph with entropy variation given in Equation (5), the best strategy to find the MRS requires the computation of all possible MRSs and the selection of that one maximizing the average value of EV. Figure 2 shows the corresponding flowchart to this algorithm. The distance from each vertex to the others is computed in a previous step. Then, all subsets of *n* vertices selected from the set of *N* graph vertices are determined. For each subset, denoted by *s*, we have to determine if it is an RS, so that our method requires the following procedure: Firstly, compute labels from the distance of each graph vertex to each vertex in *s*; secondly, determine if all labels are different, so since the number of MRSs can not be determined a priori, the algorithm finishes when all MRSs of *n* vertices are computed. For each MRS, the average value of EV is calculated and a set with the maximum value is chosen.

Moreover, we apply the algorithm proposed in [16] to find the MCDS whose vertices maximize the average value of EV. The RCDS is computed by the union of both the MRS and the MCDS. As we have explained in Section 3.1, this method does not guarantee the obtaining of the MRCDS for all graphs. This algorithm will be termed as *Approach 1*.

A considerable reduction in computational load can be reached when the subsets *s* are selected from vertices in the MCDS, instead of computing them from all vertices in the graph. This approach, termed as *Approach 2* in the following, will allow us to find the c-RS and c-MRS. Notice that the result could be the empty set.

### 3.4. Computation of an RS Based on EV

**Figure 3:**
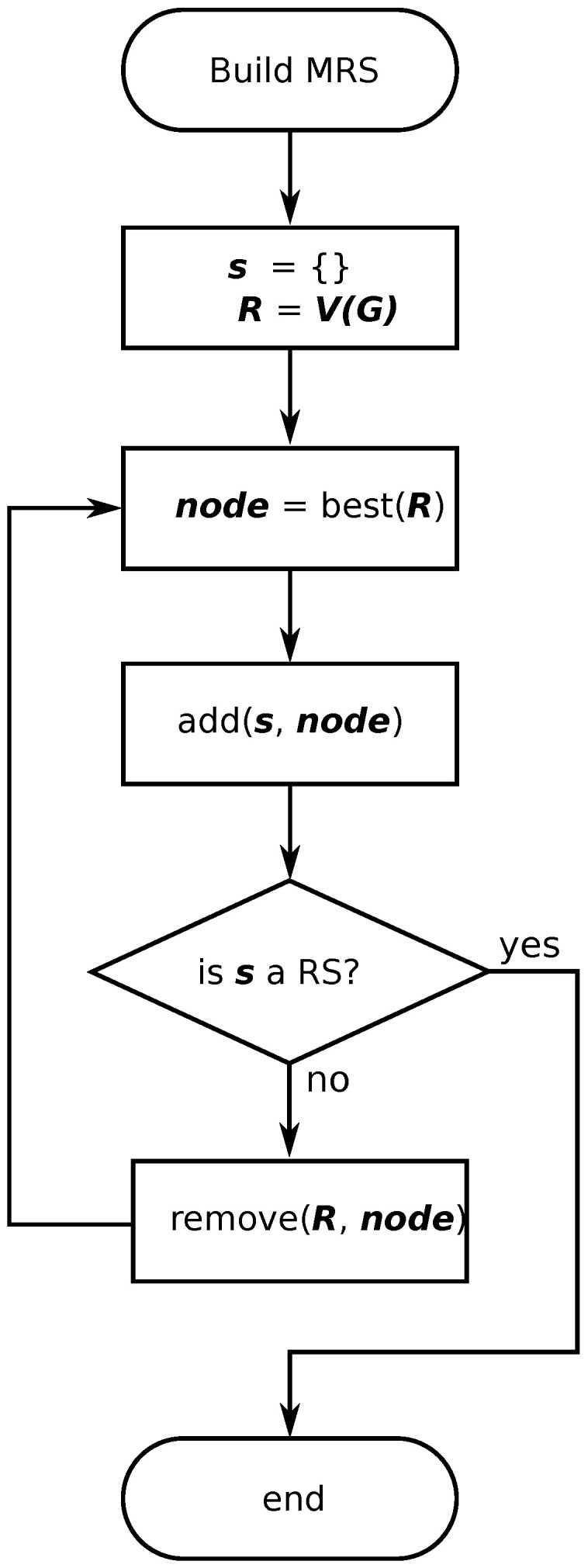
Flowchart for the computation of the resolving set (RS) according to the EV maximization.

The previous algorithms require to compute all MRSs, which is computationally expensive for large graphs. For this reason, we propose a suboptimal algorithm based on the RS computation considering the vertex with maximum EV. Figure 3 shows the corresponding flowchart to this algorithm, termed as *Approach 3*. Both the distance from each vertex to the others and the EV according to Equation (5) are computed in the previous steps. Then, the vertex with maximum EV is chosen and added to the set *s*. For this set, we have to determine if it is an RS or not. If the set *s* is not an RS, the algorithm removes from *s* the vertex with biggest EV. The algorithm finishes when an RS is found. The RCDS is computed by the union of such an RS and the MCDS obtained using the algorithm presented in [16].

## 4. Simulation Results

In this section, we will show some simulation results for comparison purposes in the performance of the three approaches proposed in Section 3.

Firstly, we consider the simple graphs mentioned in Section 3.2 there included i.e., path, cycle, and grid graphs. Remember that for these examples the MRCDS size has been already analyzed in previous works, as was explained in Proposition 1.

Then, we have performed a second set of experiments for an ad hoc wireless network under the consideration of two different configurations. For this graph type, it is not possible to exactly determine the MRCDS cardinality so Proposition 1 only establishes a minimum threshold.

### 4.1. Simple Graphs

In this subsection, we will compare the MRCDS sizes obtained from the three approaches above presented on simple graphs that are well studied in the related literature. For this purpose, we have generated 50 independent realizations varying the vertex reliability i.e., *f*. As a result, we show in Table 1 the average sizes of each approach for the path, cycle, and grid graphs of 4, 9, and 16 vertices and also the $\gamma_{rc}$ number given in Proposition 1.

From this table, for the path case, it can be said that *Approach 1* does not get the MRCDS due to the computation of the union of both MCDS and MRS, as was already shown in Section 3.2. However, *Approach 2* achieves the optimum results because it computes the c-RS. Finally, *Approach 3* exhibits good results close to the optimum ones with lower computational costs.

Observing now the results for the cycle graph, both approaches, *Approach 1* and *Approach 2*, give us the optimum values independently from the vertex number, as in both cases, it is possible to find a c-MRS, as was explained in Section 3.2. Again, it can be observed that *Approach 3* gives almost optimum sizes.

Finally, for the grid case, *Approach 1* obtains optimum sizes for 4 and 9 vertices, while *Approach 2* does not provide a solution for 9 and 16 vertices since it is not possible to find neither c-RS nor c-MRS. As happened for the other two graph types, *Approach 3* gives again a solution close to the optimum one.

### 4.2. Ad Hoc Wireless Network

An ad hoc wireless network is a decentralized type of wireless network characterized by a lack of fixed communication infrastructure where a virtual backbone is used to frequently exchange routing information (traffic conditions, neighborhood information, etc.) and broadcast messages to the network. In this section, we will present some results for the performance evaluation achieved from the application of our algorithm proposed in Section 3.3 for purposes of RCDS finding. As far as we know, the computation of the RCDS for these type of networks has been not addressed in the recent literature. Remember that the distance in Equation (1) is referred to the number of hops between vertices, not to the physical distance.

**Figure 4:**
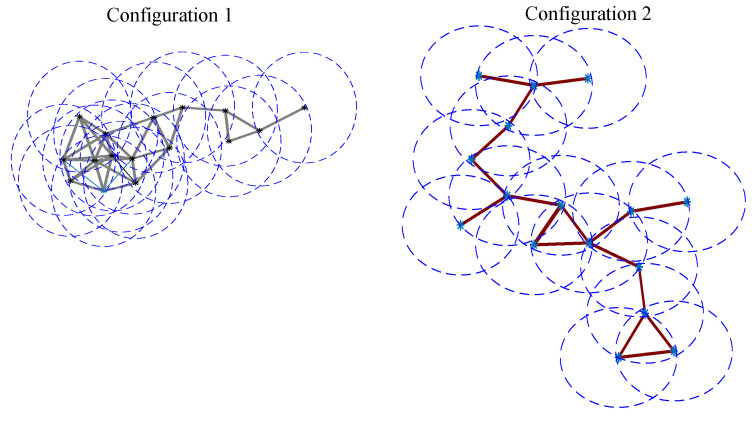
Simulation results: ad hoc sensor networks with 16 sensors.

**Figure 5:**
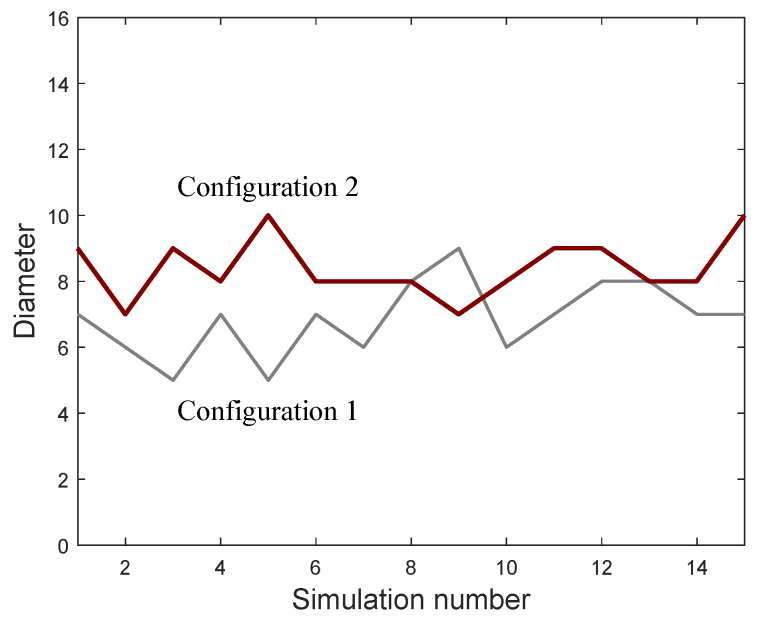
Simulation results: diameter of the ad hoc sensor networks.

We consider the Unit Disk Graph (UDG) model [12,29,30], where the network is defined by G=(V,E). The vertices in *V* are embedded in the Euclidean plane. We assume that the maximum transmission range is the same for all vertices of the network and it is unit scaled. There exists an edge u,v∈E if *u* and *v* are in the maximum transmission range of each other i.e., the Euclidean distance is d(u,v)≤1. Figure 4 shows two configurations of a UDG formed by 16 vertices, the coverage radius of each vertex and the connection between them. For each configuration, we have generated 15 independent realizations. The weight *f* has been generated following a uniform distribution and the importance of each vertex has been computed using Equation (5). Figure 5 shows the graph diameter obtained from each realization. It can be seen that, in general, the second configuration exhibits a diameter greater than the first one.

**Figure 6:**
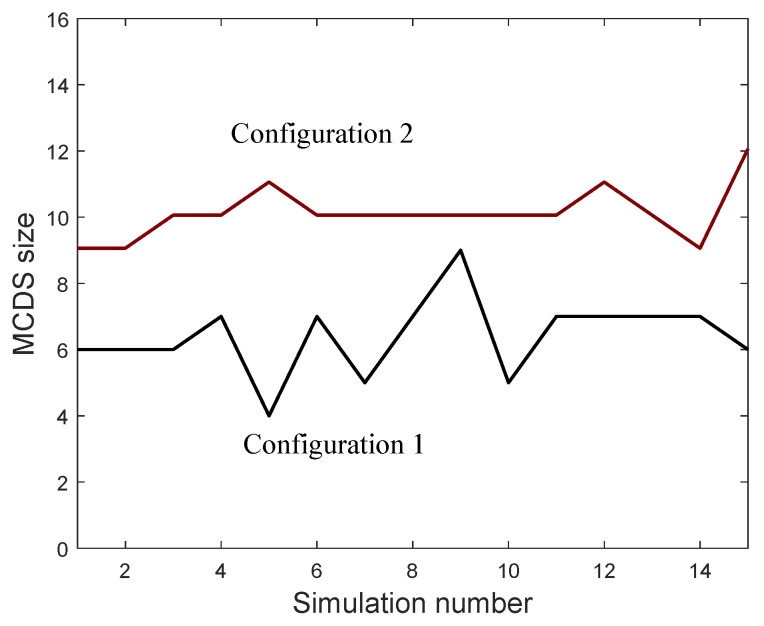
Simulation results: minimum connected dominating set (MCDS) size of the ad hoc sensor networks.

The MCDS computation has been performed using the algorithm proposed in [16], which requires one to compute both the degree and importance of each vertex. Figure 6 shows the MCDS size obtained for each realization in both network configurations. We can see that this size is bigger for Configuration 2 as its diameter is also bigger.

**Figure 7:**
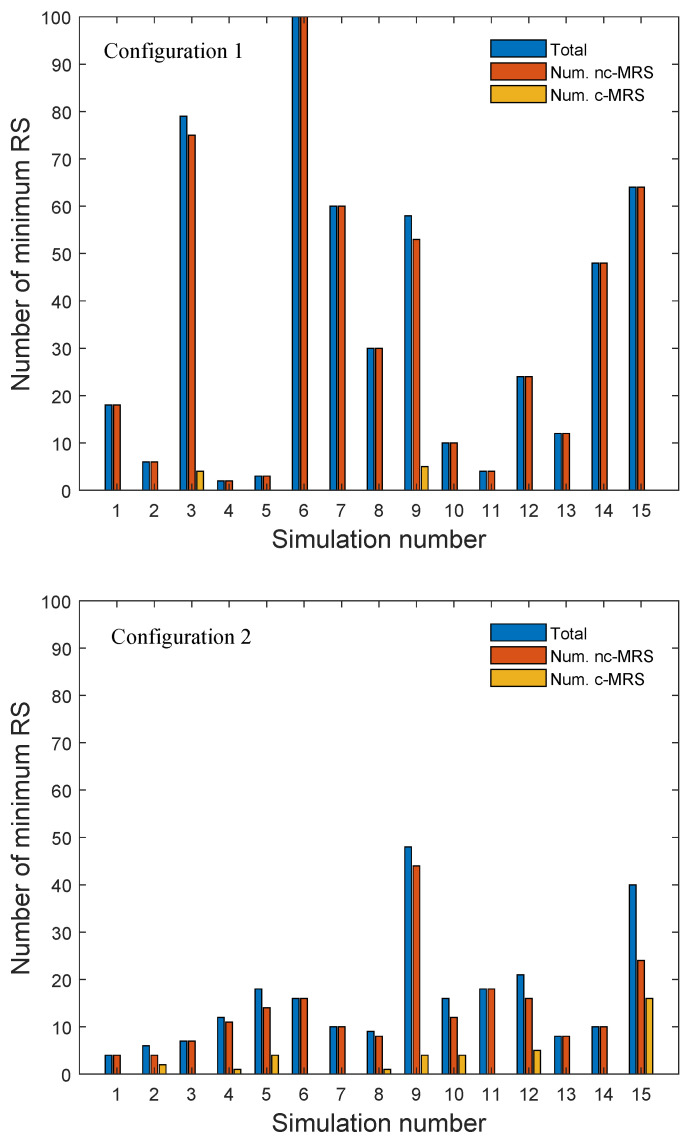
Simulation results: number of MRSs in the ad hoc sensor networks for Configuration 1 (**top**) and Configuration 2 (**bottom)**.

The computation of the MRS using the algorithm described in Figure 2 requires to calculate the distance between each pair of vertices. Figure 7 shows the number of MRSs located in the graph. This figure also shows the number of nc-MRSs and c-MRSs. Comparing the results obtained from both configurations, it can be concluded that the number of MRSs is larger for Configuration 1 although the number of c-MRSs is greater for the second one, which is a consequence of its bigger MCDS size (see Figure 6).

**Figure 8:**
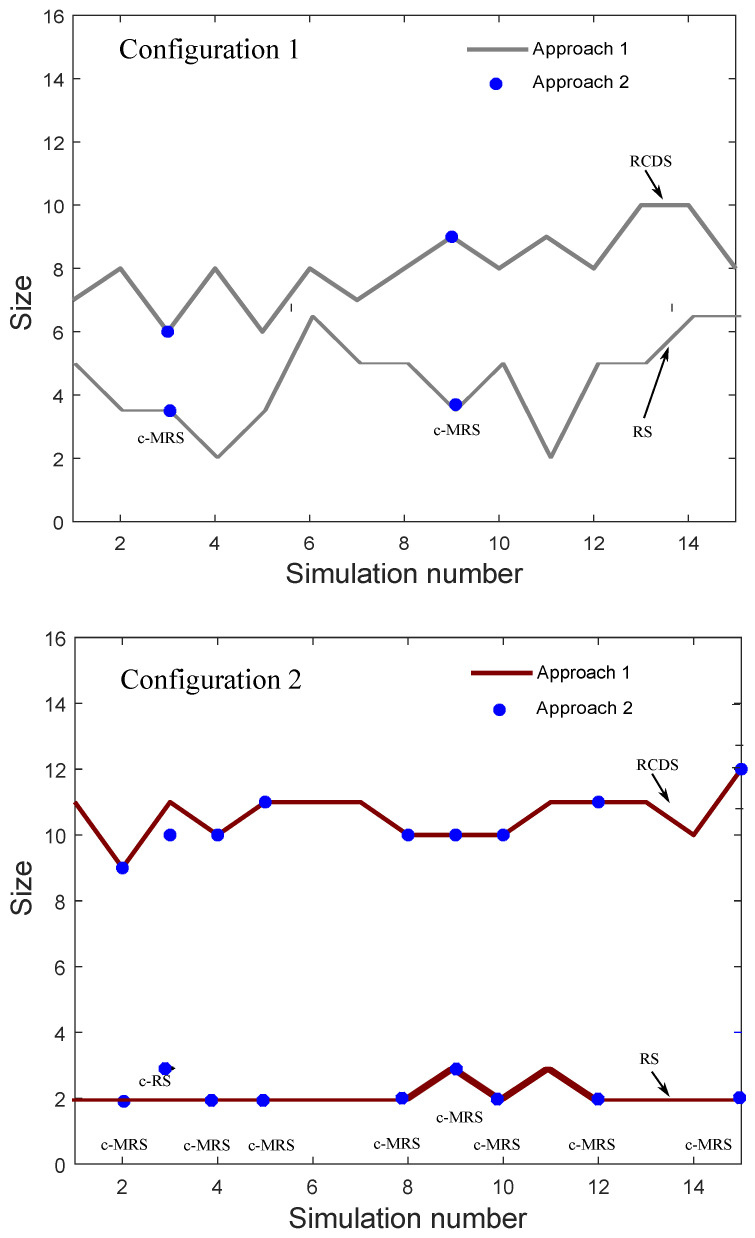
Simulation results: resolving set (RS) and resolving connected dominating set (RCDS) sizes in ad hoc sensor networks using approaches 1 and 2 for Configuration 1 (**top**) and Configuration 2 (**bottom)**.

Now, we will compare the approaches 1 and 2 presented in Section 3.3. Recall that *Approach 1* searches the MRS considering all vertices in the graph, while *Approach 2* only considers the vertices in the MCDS previously computed. Figure 8 shows both RS and RCDS sizes obtained using *Approach 1* (solid line) and *Approach 2* (dash line). This figure also marks the realizations where *Approach 2* has obtained a c-MRS or a c-RS. We can also observe that in the case of the first configuration, a c-MRS is only computed for 2 of 15 realizations. For the second configuration, *Approach 2* has found 8 c-MRSs and 1 c-RS.

**Figure 9:**
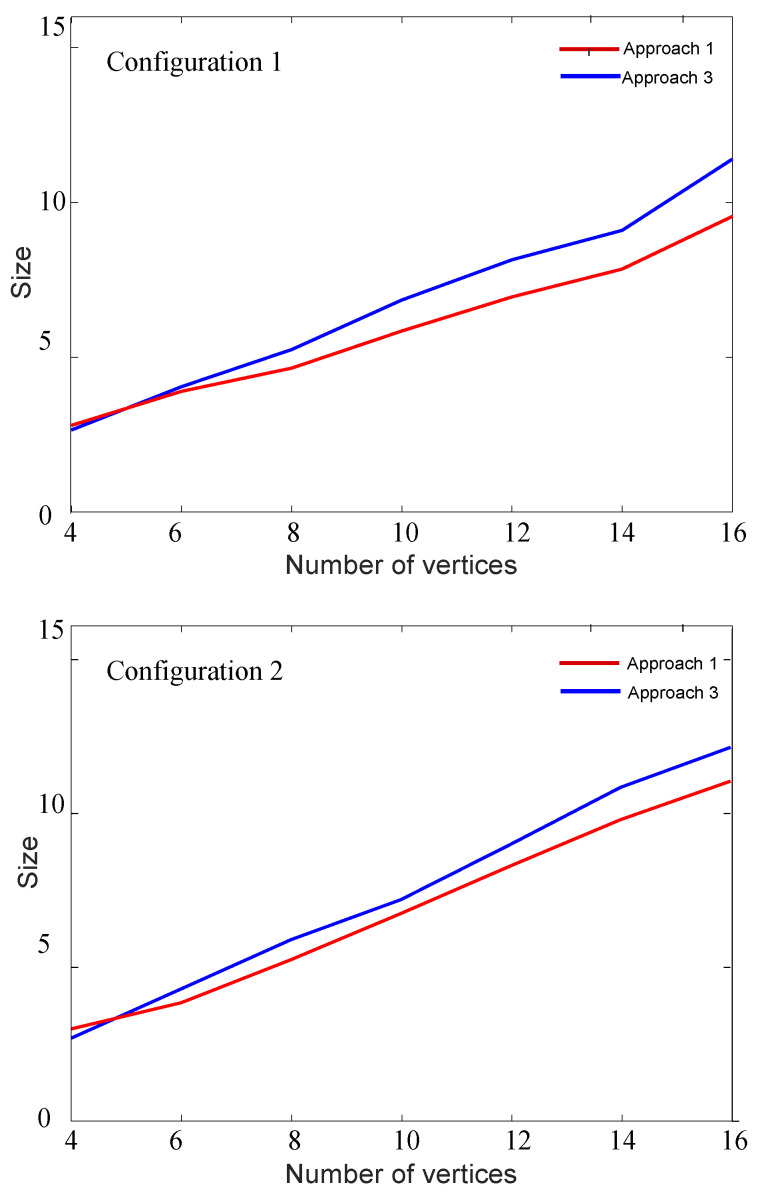
Simulation results: RCDS size in ad hoc sensor networks using approaches 1 and 3 for Configuration 1 (**top**) and Configuration 2 (**bottom)**.

Next, since *Approach 3* is an heuristic algorithm based on selecting the vertices according to the EV maximization, we are interested in comparing the RCDS sizes to those obtained from *Approach 1*. For this aim, we have generated networks from 4 to 16 vertices, and performed 15 independent realizations for different values of *f*. Figure 9 shows the RCDS size for both approaches.

### 4.3. Discussion

The computation of the MCDS has been extensively studied in the literature because it is an NP problem. We assume that the set obtained using these algorithms is a MCDS. For simulation, we have used the method described in [16] as the MCDS is computed for EV maximization.

Our algorithm that finds the RS is capable of obtaining all sets with minimum size. From these MRSs, only one is selected according to the criterion of EV maximization. Since *Approach 1* performs the union of both MCDS and MRS thus obtained, this procedure guarantees that an RCDS can be achieved. *Approach 2* looks for the RS only considering vertices in the CDS, which does not guarantee an MRS. However, the union of the RS and an MCDS is also an MRCDS.

As shown in Section 4.1, from the comparative study of all approaches on well known simple graphs, we always obtained the predictable performances for *Approach 1* and *Approach 2*, as pointed in Section 3.2, while our proposed *Approach 3* is almost optimal for all scenarios.

For an ad hoc wireless network, from Figure 8 it can be seen that, in the third realization of our simulations, the size of the RCDS obtained from *Approach 2* is smaller than that obtained from *Approach 1*. However, as can be seen from Figure 7, *Approach 2* does not guarantee an RS. In fact, this figure shows that the number of c-MRSs obtained for Configuration 2 is bigger than that obtained for Configuration 1. Observing Figure 6, we can conclude that this result is a consequence of the biggest MCDS sizes in the case of Configuration 2.

*Approach 3* is a heuristic algorithm with low computational cost based on selecting the vertices according to the EV maximization. This procedure does not guarantee an MRS. However, as happened for *Approach 1*, this algorithm provides a solution for all simulations. The results shown in Figure 9 illustrate that there is only a small increase in the RCDS size compared to that obtained from *Approach 1*.

## 5. Conclusions

We present three strategies to find the resolving connected dominating sets of a graph in wireless networks where their vertices usually have different weights. The first strategy is based on computing all the minimum resolving sets, which requires to perform a large number of searches in the graph. The computational cost of such a method can be reduced by computing the resolving set considering vertices in the minimum dominating set. However, this second approach does not guarantee to obtain a resolving set.

Finally, we have also presented a suboptimal approach based on creating the resolving set with an increasing structure. Such a set is constructed by adding the vertices according to their weights. The algorithm stops when a resolving set is found. Simulation results show that this approach gives solutions similar in performance to the optimal strategies, but with the significant advantage of considerably reducing the computational cost.

## Figures and Tables

**Table 1:** Average minimum resolving connecting dominating set (MRCDS) sizes and $\gamma_{rc}$ for simple graphs.

**(a) Path**				
**Number of Vertices**	**Approach 1**	**Approach 2**	**Approach 3**	$\gamma_{\mathrm{rc}}$
4	3	2	2.8667	2
9	8	7	7.5	7
16	15	14	14.26	14
**(b) Cycle**				
**Number of Vertices**	**Approach 1**	**Approach 2**	**Approach 3**	$\gamma_{\mathrm{rc}}$
4	2	2	2.34	2
9	7	7	7	7
16	14	14	14	14
**(c) Grid**				
**Number of Vertices**	**Approach 1**	**Approach 2**	**Approach 3**	$\gamma_{\mathrm{rc}}$
4	2	2	2.34	2
9	5	-	5.28	5
16	9.06	-	9.027	9

## Abbreviations

The following abbreviations are used in this manuscript,

CDS	Connected dominating set
c-MRS	Contained-minimum resolving set
c-RS	Contained-resolving set
DS	Dominating set
EV	Entropy variation
MCDS	Minimum connected dominating set
MRCDS	Minimum resolving connected dominating set
MRS	Minimum resolving set
nc-MRS	Non-contained-minimum resolving set
RCDS	Resolving connected dominating set
RS	Resolving set
UDG	Unit disk graph
